# A systematic review and meta-analysis of the effects of non-pharmacological interventions in improving psychological status and sleep quality among hemodialysis patients

**DOI:** 10.3389/fpubh.2026.1919741

**Published:** 2026-09-28

**Authors:** Minjie Liu, Haixia Chen, Yunyun Liu, Yaya Xi, Jing Su, Juanping Zhong, Xinman Dou, Xinglei Wang

**Affiliations:** 1 Lanzhou University School of Nursing, Lanzhou, China; 2 Department of Surgical Oncology, Lanzhou University Second Hospital, Lanzhou, China; 3 Department of Cardiac Care Unit, Lanzhou University Second Hospital, Lanzhou, China; 4 Department of Neurological Care Unit, Lanzhou University Second Hospital, Lanzhou, China; 5 Department of Nursing, Lanzhou University Second Hospital, Lanzhou, China; 6 Department of Cardiology, Lanzhou University Second Hospital, Lanzhou, China

**Keywords:** anxiety, depression, Maintenance hemodialysis, meta-analysis, non-pharmacological interventions, sleep quality, systematic review

## Abstract

**Background:**

Patients receiving maintenance hemodialysis **(MHD)** experience substantial physical and psychological burdens. Non-pharmacological interventions have been increasingly explored as potential approaches for improving psychological symptoms and sleep quality in this population.

**Objective:**

To systematically evaluate the effects of non-pharmacological interventions on depression, anxiety, and sleep quality in patients receiving MHD.

**Methods:**

Systematic searches were conducted in PubMed, Embase, CINAHL Complete, Cochrane Library, and Web of Science from January 2000 to February 2026 for English-language randomized controlled trials **(RCTs)**. The Cochrane Risk of Bias 2 **(RoB 2)** tool was used to assess the risk of bias in included trials. Standardized mean differences **(SMDs)** with 95% confidence intervals **(CIs)** were used as effect estimates for continuous outcomes. The Grading of Recommendations Assessment, Development and Evaluation **(GRADE)** approach was used to assess the certainty of evidence. Meta-analyses were performed using RevMan 5.4 and R version 4.5.2.

**Result:**

Fourteen randomized controlled trials were included. Meta-analyses comprised 9 studies (570 participants) for depression, 11 studies (647 participants) for anxiety, and 14 studies (871 participants) for sleep quality. Compared with usual care, non-pharmacological interventions significantly improved depression (SMD = −0.62, 95% CI: −0.87 to −0.37, I^2^ = 52%), anxiety (SMD = −0.86, 95% CI: −1.23 to −0.49, I^2^ = 79%), and sleep quality (SMD = −0.94, 95% CI: −1.33 to −0.55, I^2^ = 87%). Leave-one-out sensitivity analyses did not materially reduce heterogeneity for anxiety or sleep quality. Exploratory subgroup analyses revealed no statistically significant differences in effect estimates according to intervention type or duration (*P* > 0.05). Potential publication bias was suggested for depression, whereas no statistically significant evidence of publication bias was identified for anxiety or sleep quality. Based on the GRADE framework, the certainty of evidence was moderate for depression and low for anxiety and sleep quality.

**Conclusion:**

The available evidence suggests that non-pharmacological interventions may improve depressive symptoms, anxiety symptoms, and sleep quality in patients receiving MHD. However, the certainty of evidence was low for anxiety and sleep quality outcomes and moderate for depressive symptoms, and these findings should be interpreted cautiously.

**Systematic Review Registration:**

Identifier CRD420261370746.

## 1 Introduction

Chronic kidney disease **(CKD)** is a major global public health challenge, affecting approximately 780 million adults and causing 1.48 million deaths worldwide in 2023 (1). CKD is classified into five stages according to glomerular filtration rate **(GFR)**. End-stage renal disease **(ESRD)**, also known as uremia, is diagnosed when GFR falls below 15 mL/(min·1.73 m^2^). It is estimated that approximately 2% of patients with CKD progress to ESRD worldwide each year (2). The primary treatment options for ESRD include kidney transplantation and dialysis. Kidney transplantation can restore kidney function and is associated with better long-term survival and quality of life than dialysis, and is therefore regarded as the preferred treatment option for eligible patients (3, 4). However, access to kidney transplantation remains limited by the risk of transplant-related infections and substantial disparities in healthcare resources and treatment availability, particularly in low- and middle-income countries (5, 6). Globally, dialysis accounts for 78% of all renal replacement therapies, of which 89% is hemodialysis and 11% is peritoneal dialysis (7). MHD serves as a life-saving core therapy by simulating the filtration function of healthy kidneys to remove toxins, excess fluid, and metabolic waste from the bloodstream (8).

Although survival among patients patients receiving MHD has improved, contemporary healthcare increasingly emphasizes outcomes beyond physical survival, including psychological wellbeing and quality of life (9). Psychological and sleep-related problems among patients receiving MHD include depression, anxiety, poor sleep quality, and cognitive impairment. These problems can substantially impair quality of life and may be associated with adverse clinical outcomes, including hospitalization and, in some patients, suicidal ideation. Depressive and anxiety symptoms are highly prevalent among patients receiving dialysis, with reported prevalence ranges of 37%−42% and 38%−53%, respectively (10). Another study reported a prevalence of anxiety symptoms of 57.7% among patients receiving dialysis (11). Previous studies have demonstrated that the number and severity of complications in MHD patients are significantly associated with depressive symptoms (12). The psychological burden associated with MHD may extend beyond patients themselves to their family caregivers, whose psychological resilience can be affected by the demands of long-term caregiving (13). Poor sleep quality affects 64.2% of patients undergoing MHD worldwide (14). Studies have indicated that poor sleep quality may further aggravate physical discomfort and contribute to a vicious cycle with anxiety and depression through bidirectional interactions (15). The complex interplay among depression, anxiety, and poor sleep quality may further increase the burden on patients and their families and underscores the need for comprehensive nursing assessment, individualized interventions, and ongoing psychological care, highlighting the importance of targeted intervention research.

Currently, pharmacotherapy remains the mainstay for depression in MHD patients in clinical practice. However, MHD patients present with renal impairment, which limits the excretion of conventional antidepressants metabolized primarily by the kidneys, and high-quality clinical evidence is scarce (16). Affected by the unique physiological status of dialysis patients, the safety and efficacy of these medications remain highly controversial, and long-term administration carries potential risks of adverse reactions (17, 18). Accordingly, non-pharmacological interventions have gradually become a key research direction in this field. For anxiety management, existing studies prefer non-pharmacological interventions. For example, cognitive behavioral therapy may help modify maladaptive cognitions and behaviors, thereby reducing negative emotions and psychological distress (19). Rational emotive behavior therapy may alleviate anxiety by modifying maladaptive beliefs and promoting more rational cognitive responses to stressful events (20). Pharmacological treatment remains a commonly used approach for improving sleep quality (21). Nevertheless, a growing body of evidence indicates that non-pharmacological approaches such as exercise are safe and effective. For instance, Baduanjin can improve insomnia (22), and aerobic exercise may improve sleep-related outcomes in patients undergoing hemodialysis (23). Collectively, MHD patients experience a high burden of complex psychological and sleep problems, which may interact with and exacerbate each other. Pharmacological management of sleep disturbances remains challenging because evidence regarding treatment efficacy and safety in this population is limited, with concerns regarding potential adverse effects. Consequently, pharmacotherapy alone may be insufficient to meet the long-term clinical needs of MHD patients. Therefore, developing safe, effective, and well-tolerated non-pharmacological intervention strategies is an important priority for improving psychological and sleep outcomes and optimizing the comprehensive management of MHD patients (24, 25).

Although non-pharmacological interventions have been increasingly explored, relevant existing studies still possess certain limitations. Existing systematic reviews and meta-analyses have focused on the independent effects of a single type of non-pharmacological intervention on sleep quality or depression (26), or on the effects of non-pharmacological interventions on anxiety and depression (27), or the effects of non-pharmacological interventions on sleep quality among patients receiving hemodialysis (28). However, evidence regarding the effects of diverse non-pharmacological interventions on multiple psychological and sleep-related outcomes among patients with MHD remains limited.

Therefore, this study aimed to comprehensively evaluate the effects of non-pharmacological interventions on depression, anxiety, and sleep quality in MHD patients, with each outcome analyzed separately. The findings may provide evidence to support clinical decision-making and the development of individualized nursing strategies.

## 2 Materials and methods

### 2.1 Study registration

This systematic review was prospectively registered in the International Prospective Register of Systematic Reviews **(PROSPERO)** under registration number CRD420261370746. The reporting adhered to the Preferred Reporting Items for Systematic Reviews and Meta-Analyses **(PRISMA)** guidelines (29) and the recommendations outlined in the Cochrane Handbook for Systematic Reviews (30). The study protocol was publicly available on the PROSPERO platform.

### 2.2 Literature search strategy

A comprehensive literature search was performed across five electronic databases: PubMed, Embase, CINAHL Complete, Cochrane Library, and Web of Science Core Collection. The literature search spanned from the inception of each database to February 2026. Appropriate truncation symbols and Boolean operators were used to combine five core keyword groups in titles and abstracts: MHD patients, non-pharmacological interventions, depression, anxiety, and sleep quality. Reference lists of included studies were also manually screened to identify potentially eligible additional studies. The search strategy was developed by two authors (Minjie Liu and Haixia Chen) and independently reviewed by two other authors (Yunyun Liu and Yaya Xi). Only peer-reviewed English-language articles were included. Details are presented in Supplementary material.

### 2.3 Literature inclusion and exclusion criteria

#### 2.3.1 Inclusion criteria

Inclusion criteria were established based on the PICOS framework: (1) Participants: Adults aged ≥18 years with CKD stage 5 or ESRD who were receiving regular MHD 2–3 times per week and had a dialysis vintage ≥3 months; There were no restrictions regarding gender or primary renal disease. (2) Interventions: Non-pharmacological interventions; There were no restrictions on intervention duration, frequency, or delivery format. (3) Comparators: Routine care; Wait-list control; Placebo/sham intervention. (4) Outcome measures: Studies were required to report both depression and sleep quality, or both anxiety and sleep quality, as outcomes assessed using validated scales. (5) Study design: RCTs.

#### 2.3.2 Exclusion criteria

(1) Studies without available full texts or from which valid statistical parameters could not be extracted. (2) Studies published in languages other than English. (3) Animal experiments, conference abstracts, and gray literature. (4) Studies involving pharmacological interventions or pharmacotherapy combined with non-pharmacological interventions. (5) Duplicate publications or overlapping datasets.

### 2.4 Literature screening and data extraction

After the systematic search, all retrieved records were imported into EndNote X9 for deduplication. Two researchers independently conducted the literature screening. Initially, they screened titles and abstracts for eligibility, and they subsequently reviewed the full texts of potentially relevant studies for final inclusion. Any disagreements were resolved through discussion or by consulting a third reviewer. One reviewer performed data extraction, entering all extracted information into an Excel spreadsheet, which was then cross-checked by a second reviewer. The extracted data included the first author, year of publication, sample size, baseline characteristics, intervention details, control details, intervention duration, and outcome measures for depression, anxiety, and sleep quality.

### 2.5 Assessment of methodological quality (risk of bias)

The risk of bias in RCTs was assessed using the RoB 2 tool (31). RoB 2 evaluates five key domains: bias arising from the randomization process, bias due to deviations from intended interventions, bias due to missing outcome data, bias in measurement of the outcome, and bias in selection of the reported result. Each domain was rated as “low risk of bias,” “some concerns,” or “high risk of bias.” Two reviewers independently conducted the assessments, and any disagreements during the assessment were resolved through discussion or consultation with a third reviewer.

### 2.6 Data synthesis and statistical analysis

Continuous outcome data, including means, standard deviations, and sample sizes of intervention and control groups, were extracted from eligible studies. For continuous outcomes assessed using different measurement instruments, differences in measurement scales and scoring ranges precluded direct comparison of raw mean differences across instruments. Therefore, SMDs with 95% CIs were used as pooled effect estimates. Before meta-analysis, the direction of scoring was standardized across measurement instruments so that higher scores consistently indicated more severe symptoms or poorer sleep quality. For studies with multiple intervention arms compared with a common control group, only one intervention arm was included to avoid double-counting of participants. For crossover trials, only data from the final intervention period were extracted unless combined results were reported by the original authors. Outcomes reported in non-standard formats were converted into SMDs and standard errors when possible; otherwise, they were summarized descriptively. Between-study heterogeneity was quantitatively assessed using *P* value and I^2^ statistic, representing the proportion of total variation attributable to between-study heterogeneity rather than sampling error. I^2^ values were interpreted as follows: ≤ 50% indicated low heterogeneity, 50%−75% indicated moderate heterogeneity, and >75% indicated substantial heterogeneity. The I^2^ statistic was used solely to quantify the magnitude of statistical heterogeneity and was not interpreted as an indicator of clinical or methodological heterogeneity. Given the clinical and methodological heterogeneity across the included studies, arising from variations in non-pharmacological intervention modalities, intervention duration and protocols, delivery modes, participant characteristics, control conditions, and outcome assessment instruments, random-effects models were used for all meta-analyses. Sensitivity analyses were conducted to assess the robustness of the pooled estimates. A leave-one-out approach was performed by sequentially excluding individual studies to examine their influence on the pooled effect estimates and heterogeneity. In addition, a *post-hoc* exploratory multiple-study exclusion analysis was conducted to further explore potential sources of heterogeneity (32).

Given the common clinical objective of improving psychological symptoms and sleep quality among patients receiving MHD, non-pharmacological interventions were first synthesized as a broad intervention category, while recognizing that individual modalities may differ in their therapeutic components and mechanisms. Subgroup analyses were performed according to intervention modality to explore potential sources of heterogeneity. Given the limited number of studies in some subgroups, these analyses were considered exploratory. Non-pharmacological interventions were categorized into five groups based on their primary therapeutic components: (1) exercise-based interventions, centered on physical activity and potentially improving cardiovascular and other physiological functions (33); (2) cognitive behavioral interventions, targeting maladaptive cognitions and behaviors through cognitive restructuring and behavioral modification (34); (3) music relaxation interventions, using music-related auditory stimulation to promote relaxation and emotional regulation (35); (4) mind–body relaxation interventions, using active mind–body self-regulation to reduce psychological and physiological arousal (36); (5) complementary and traditional medicine-related interventions, involving somatic or sensory stimulation and other traditional therapeutic approaches (37). Intervention duration was categorized as ≤ 4 weeks or >4 weeks based on the distribution of intervention durations across the included studies. Potential small-study effects were assessed by visual inspection of funnel plots, and Egger's regression test was conducted to evaluate potential publication bias when at least 10 studies were available for a given outcome (38). In interpreting the results of the meta-analysis, Cohen's criteria were used to interpret the magnitude of SMDs (39) with absolute SMD values of 0.2, 0.5, and 0.8 representing small, moderate, and large effects, respectively.

### 2.7 Quality of evidence assessment

The updated GRADE system (40) was applied to assess the certainty of evidence using four grades: high, moderate, low, and very low. The low and very low certainty of evidence means that the true effect is likely to be substantially different from the estimate of effect, and we have little or very little confidence in the effect estimate. Any discrepancy about grading the certainty of the evidence was resolved through discussion with the corresponding author.

## 3 Results

### 3.1 Study selection and characteristics of included studies

A total of 951 records were identified through literature searches. After removing 128 duplicates, 823 records remained for title and abstract screening. Following screening against the predefined eligibility criteria, 788 records were excluded, and 35 articles underwent full-text assessment. After full-text review, 21 studies were excluded, resulting in 14 RCTs being included in the meta-analysis. The study selection process is presented in Figure 1.

**Figure 1:**
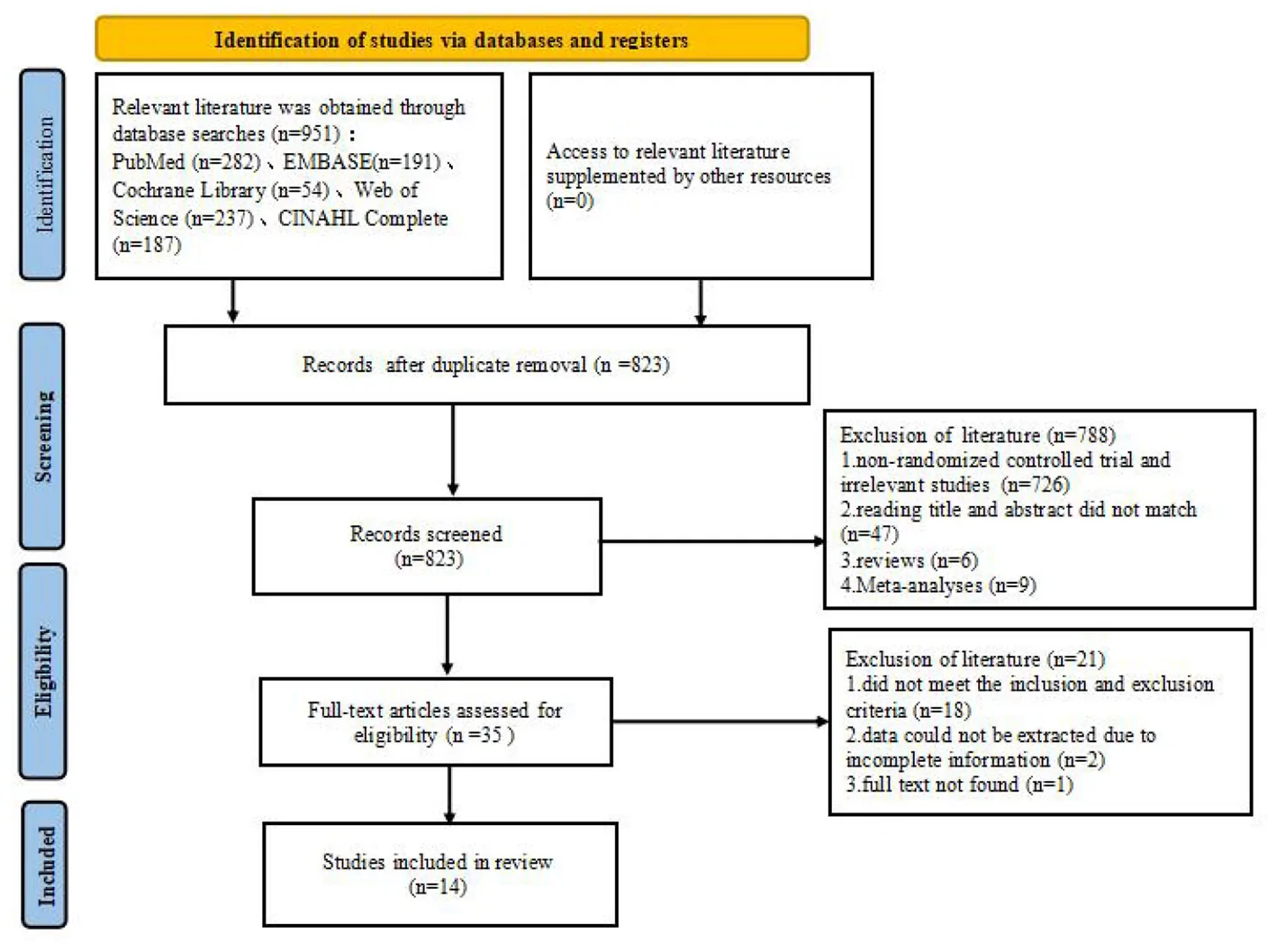
PRISMA flow chart.

A total of 14 RCTs (41–54) involving 871 participants were included in this study. Regarding geographical distribution, seven studies (41, 42, 44, 49, 51, 52, 54) were conducted in China, three (46, 48, 53) in Iran, one (43) in Turkey, two (45, 50) in Indonesia, and one (47) in Italy. For baseline characteristics, ten studies (41–46, 48, 50–52) reported age as mean ± standard deviation for both groups, two studies (47, 54) reported overall mean ± standard deviation, and one study (53) reported the mean age of participants. One study (49) did not present baseline data on age. Concerning intervention types, two studies (41, 51) employed exercise-based interventions, two studies (46, 54) utilized cognitive-behavioral interventions, two studies (42, 47) implemented music-relaxation interventions, four studies (44, 45, 48, 49) adopted mind-body relaxation interventions, and four studies (43, 50, 52, 53) applied complementary and traditional medicine-related interventions. In terms of intervention duration, seven studies (43, 45, 48–50, 52, 53) lasted 4 weeks or less, while the remaining seven studies (41, 42, 44, 46, 47, 51, 54) extended beyond 4 weeks. For depression assessment tools, one study (41) employed the Self-Rating Depression Scale **(SDS)**, four studies (42, 44, 47, 50) utilized the Depression subscale of the Hospital Anxiety and Depression Scale **(HADS-D)**, one study (51) applied the Hamilton Depression Rating Scale **(HAMD)**, and four studies (46, 49, 52, 54) implemented the Beck Depression Inventory **(BDI/BDI-II)**. Regarding anxiety assessment tools, one study (41) used the Self-Rating Anxiety Scale **(SAS)**, four studies (42, 44, 47, 50) applied the Anxiety subscale of the Hospital Anxiety and Depression Scale **(HADS-A)**, two studies (45, 51) utilized the Hamilton Anxiety Rating Scale [HAMA; referred to as HARS in (45)], two studies (46, 54) employed the Beck Anxiety Inventory **(BAI)**, and three studies (43, 48, 53) used the State-Trait Anxiety Inventory-State version **(STAI-S)**. Two scales assessed sleep quality: thirteen studies (41, 42, 44–54) adopted the Pittsburgh Sleep Quality Index **(PSQI)**, while one study (43) utilized the Richards-Campbell Sleep Questionnaire **(RCSQ)**. Among the included studies, eight (41, 42, 44, 46, 47, 50, 51, 54) reported all three outcomes (depression, anxiety, and sleep quality), four studies (43, 45, 48, 53) reported only anxiety and sleep quality, and two studies (49, 52) reported only depression and sleep quality. Thus, all included studies assessed at least one psychological outcome (depression or anxiety) in combination with sleep quality, consistent with the predefined eligibility criteria (Supplementary Table S1). The characteristics of the included studies are summarized in Table 1.

**Table 1:** Baseline characteristics of included studies.

Included studies	Country	Sample size		Age/(years, x ± s)		Interventions	Control interventions	Treatment duration	Outcome measures
		Intervention group	Control group	Intervention group	Control group				
Li et al. (41)	China	29	30	42.52 ± 10.63	46.83 ± 10.87	Aerobic combined with resistance exercise	Usual care	12	SDS; SAS; PSQI
Zhang et al. (42)	China	29	29	54.76 ± 13.00	57.52 ± 12.19	Western music	Usual care	8	HADS-D; HADS-A; PSQI
Göktuna et al. (43)	Turkey	23	22	64.39 ± 12.42	69.95 ± 8.70	Foot reflexology massage	Usual care	3	STAI-S; RCSQ
Tang et al. (44)	China	32	26	64.25 ± 5.68	67.65 ± 7.70	Emotional Freedom Techniques	Usual care	12	HADS-D; HADS-A; PSQI
Wijayanti et al. (45)	Indonesia	26	27	45.58 ± 2.176	46.56 ±1.969	Religion-based spiritual audio intervention	Usual care	2	HARS; PSQI
Shareh et al. (46)	Iran	56	57	43.7 ± 6.4	46.4 ± 7.9	Group cognitive behavioral therapy for insomnia	Usual care	9	BDI-II; BAI; PSQI
Burrai et al. (47)	Italy	12	12	62.3 ± 2.8		Live singing	Usual care	5	HADS-D; HADS-A; PSQI
Afshar et al. (48)	Iran	35	35	51.91 ± 13.88	49.28 ± 12.46	Guided imagery	Usual care	4	STAI-S; PSQI
Tsai et al. (49)	China, Taiwan Province of	32	25	Not reported		Breathing training group	Waitlist	4	BDI-II; PSQI
Suandika et al. (50)	Indonesia	49	47	45.42 ± 13.29	49.76 ± 13.18	True acupressure	Usual care	4	HADS-D; HADS-A; PSQI
Feng et al. (51)	China	28	30	57.50 ± 11.16	56.10 ± 12.07	Resistance band exercise	Usual care	12	HAMD; HAMA; PSQI
Tsay et al. (52)	China, Taiwan Province of	35	36	57.23 ± 10.93	56.81 ± 13.30	Acupressure	Usual care	4	BDI; PSQI
Heirati et al. (53)	Iran	20	17	Mean age: 55.19		Aromatherapy group	Usual care	4	STAI-S; PSQI
Chen et al. (54)	China, Taiwan Province of	37	35	Mean age: 58 ± 11		Cognitive behavioral therapy	Usual care	6	BDI; BAI; PSQI

### 3.2 Quality assessment (risk of bias) of included studies

The risk of bias of the 14 included randomized controlled trials was assessed using the Cochrane RoB 2 tool. No study was judged to have an overall low risk of bias; 12 studies had some concerns, and 2 studies were judged to have a high risk of bias (Supplementary Figures S1, S2). Across individual domains, for the randomization process, 5 studies were judged as low risk and 9 had some concerns; for deviations from intended interventions, 13 studies had some concerns and 1 was judged as high risk; for missing outcome data, 8 studies were judged as low risk and 6 had some concerns; for measurement of the outcome, 1 study was judged as low risk, 12 studies had some concerns and 1 was judged as high risk; for selection of the reported result, 1 study was judged as low risk and 13 had some concerns. Detailed domain-level judgments for each study are presented in Supplementary Table S2.

### 3.3 Meta-analysis results

#### 3.3.1 Meta-analysis of depression

Of the 14 eligible RCTs included in the present meta-analysis, 10 studies (41, 42, 44, 46, 47, 49–52, 54) assessed depression-related outcomes. One study (50) was excluded from the quantitative synthesis because the reported data could not be converted to a format suitable for calculating an SMD. Descriptively, this study reported greater improvement in anxiety symptoms in the intervention group than in the control group, which was directionally consistent with the pooled finding. The remaining nine studies (41, 42, 44, 46, 47, 49, 51, 52, 54), comprising a total of 570 participants, were included in the quantitative synthesis. Given the variability in depression assessment instruments across studies, SMDs were used as the summary effect measure to facilitate cross-study comparability. The pooled analysis indicated that non-pharmacological interventions significantly reduced depressive symptom scores compared with control conditions (SMD = −0.62, 95% CI: −0.87 to −0.37, *P* < 0.00001). Moderate statistical heterogeneity was observed across studies (I^2^ = 52%, *P* = 0.03). In addition to statistical heterogeneity, clinical heterogeneity was evident in intervention modalities, intervention duration, delivery formats, control conditions, depression assessment instruments, and participants' baseline characteristics. Therefore, a random-effects model was used for the meta-analysis (Figure 2). A leave-one-out sensitivity analysis was conducted to assess the influence of individual studies on the pooled estimate and heterogeneity (Supplementary Table S3). Exclusion of study (44) reduced the observed heterogeneity, with I^2^ decreasing from 52% to 38% (*P* = 0.13) among the remaining eight studies (41, 42, 46, 47, 49, 51, 52, 54) The pooled effect remained statistically significant (SMD = −0.54, 95% CI: −0.77 to −0.31, *P* < 0.00001), with the direction of effect consistent with that of the primary analysis (Figure 3).

**Figure 2:**
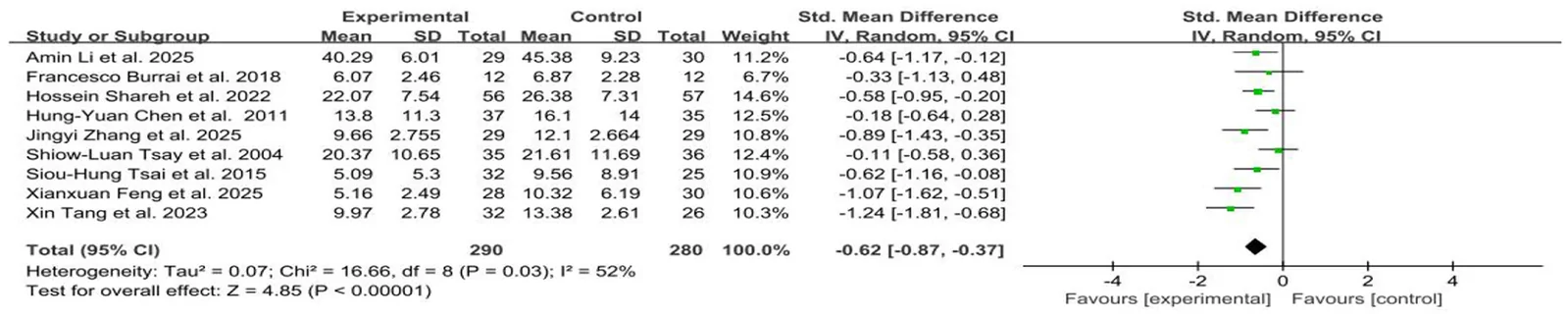
Meta-analysis of depressive symptoms based on 9 studies of non-pharmacological interventions in patients receiving MHD.

**Figure 3:**
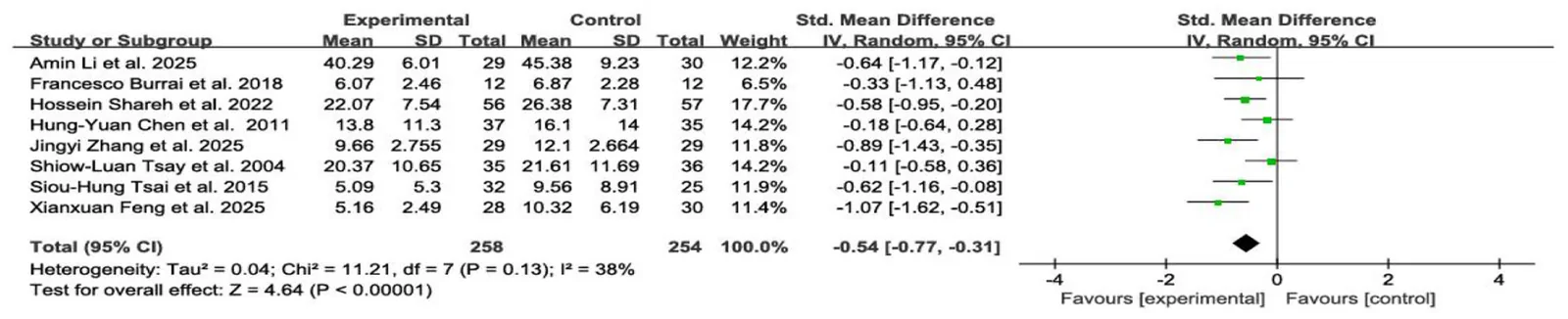
Sensitivity analysis of depressive symptoms based on 8 studies of non-pharmacological interventions in patients receiving MHD.

#### 3.3.2 Meta-analysis of anxiety

Of the 14 eligible RCTs included in the present meta-analysis, 12 studies (41–48, 50, 51, 53, 54) reported anxiety-related outcomes. One study (50) was excluded from the quantitative synthesis because the reported data could not be converted to a format suitable for calculating an SMD. Descriptively, this study reported greater improvement in anxiety symptoms in the intervention group than in the control group, which was directionally consistent with the pooled finding. The remaining 11 studies (41–48, 51, 53, 54), comprising a total of 647 participants, were included in the quantitative synthesis. Given the use of different anxiety assessment instruments across studies, SMDs were used as the summary effect measure to enhance comparability across studies. The pooled analysis indicated that non-pharmacological interventions significantly reduced anxiety symptom scores compared with control conditions (SMD = −0.86, 95% CI: −1.23 to −0.49, *P* < 0.00001). High statistical heterogeneity was observed across the included studies (I^2^ = 79%, *P* < 0.00001). In addition to statistical heterogeneity, clinical heterogeneity was evident in intervention modalities, intervention duration, delivery formats, control conditions, anxiety assessment instruments, and participants' baseline characteristics. Therefore, a random-effects model was used for the primary meta-analysis (Figure 4). To assess the influence of individual studies on the pooled effect estimate and heterogeneity, a leave-one-out sensitivity analysis was conducted (Supplementary Table S4). A *post-hoc* exploratory sensitivity analysis excluding studies (45, 48) was subsequently conducted, which reduced the observed heterogeneity among the remaining nine studies (41–44, 46, 47, 51, 53, 54) with I^2^ decreasing from 79% to 7% (*P* = 0.38). Re-pooling the remaining studies using the random-effects model yielded a statistically significant reduction in anxiety symptom scores (SMD = −0.59, 95% CI: −0.78 to −0.41, *P* < 0.00001), with the direction of effect consistent with that of the primary analysis (Figure 5).

**Figure 4:**
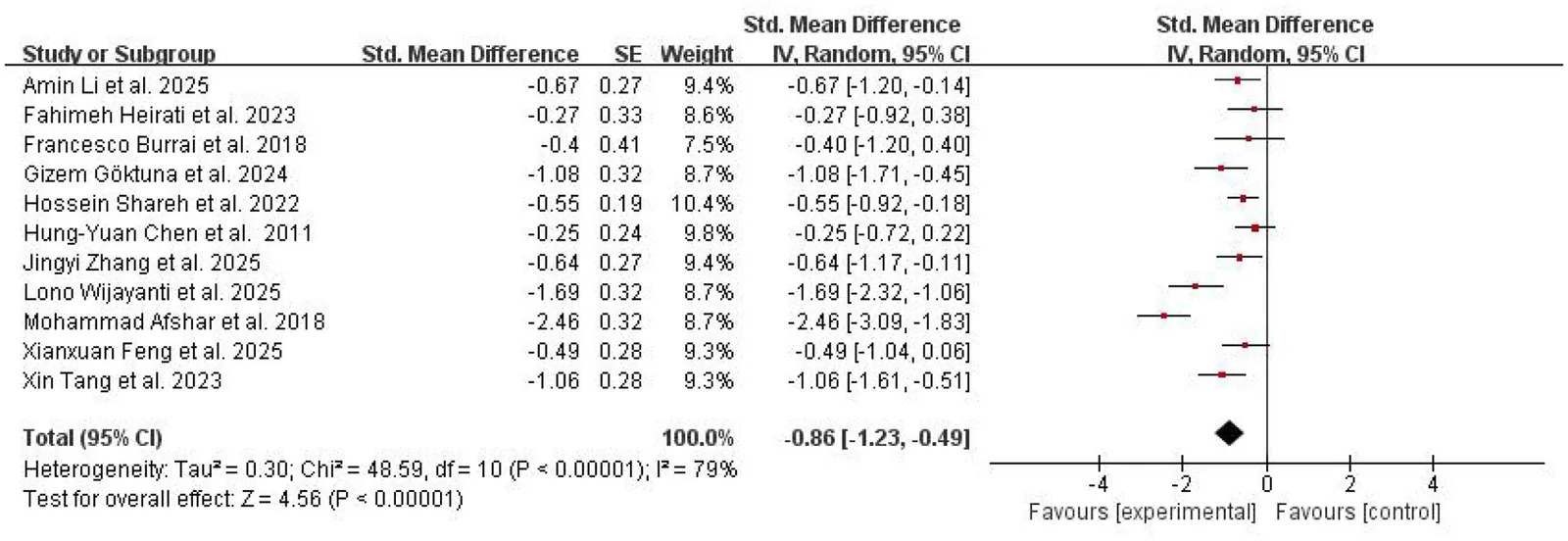
Meta-analysis of anxiety symptoms based on 11 studies of non-pharmacological interventions in patients receiving MHD.

**Figure 5:**
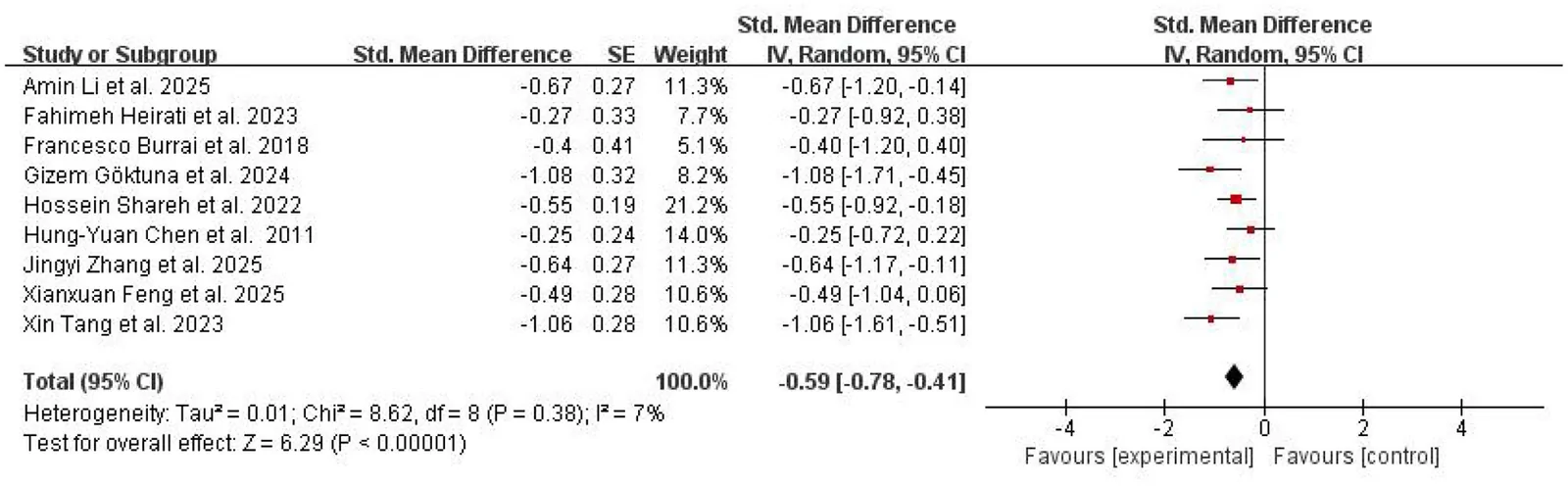
Sensitivity analysis of anxiety symptoms based on 9 studies of non-pharmacological interventions in patients receiving MHD.

#### 3.3.3 Meta-analysis of sleep quality

All 14 randomized controlled trials included in this review reported outcomes related to sleep quality. Because different sleep quality assessment instruments were used across studies, the SMD was used as the pooled effect measure. The pooled analysis indicated significantly improved sleep quality in the intervention group compared with the control group, with a large pooled effect size (SMD = −0.94, 95% CI: −1.33 to −0.55, *P* < 0.00001). Substantial statistical heterogeneity was observed across studies (I^2^ = 87%, *P* < 0.00001). In addition to statistical heterogeneity, clinical heterogeneity was also present across studies, mainly in terms of intervention type, intervention duration, delivery mode, control conditions, sleep assessment instruments, and participant baseline characteristics (Figure 6). To assess the influence of individual studies on the pooled effect estimate and heterogeneity, a leave-one-out sensitivity analysis was first performed (Supplementary Table S5). Removal of any single study did not substantially reduce the observed heterogeneity. A *post-hoc* exploratory analysis was then conducted by simultaneously excluding four studies (41, 43, 48, 50), which were identified based on visual inspection of the forest plot and consideration of potential clinical and methodological differences across studies. After simultaneously excluding these four studies, heterogeneity among the remaining 10 studies (42, 44–47, 49, 51–54) decreased to a moderate level (I^2^ = 38%, *P* = 0.11), and the pooled effect remained statistically significant but was attenuated in magnitude (SMD = −0.56, 95% CI: −0.77 to −0.34, *P* < 0.00001; Figure 7). The direction of the effect remained consistent with the primary analysis, whereas the attenuation of the pooled effect suggested that these four studies may have influenced both the observed heterogeneity and the magnitude of the pooled effect.

**Figure 6:**
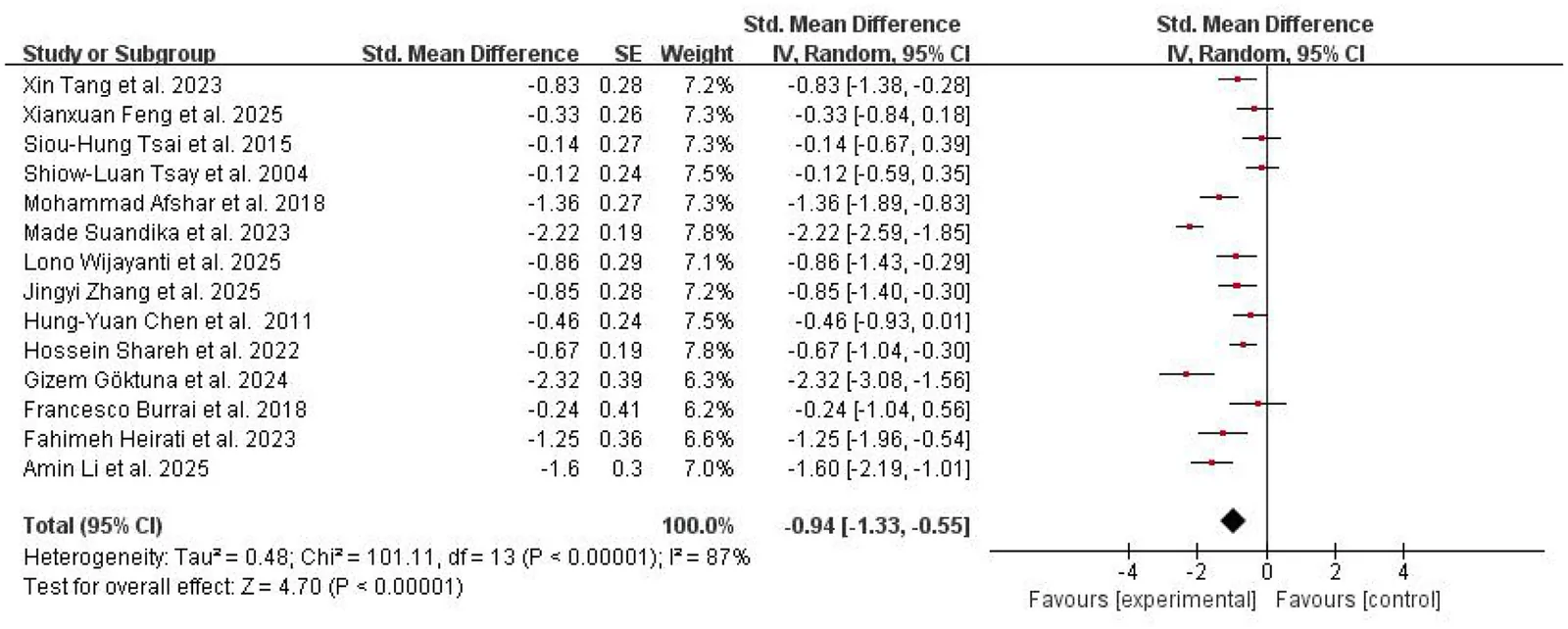
Meta-analysis of sleep quality based on 14 studies of non-pharmacological interventions in patients receiving MHD.

**Figure 7:**
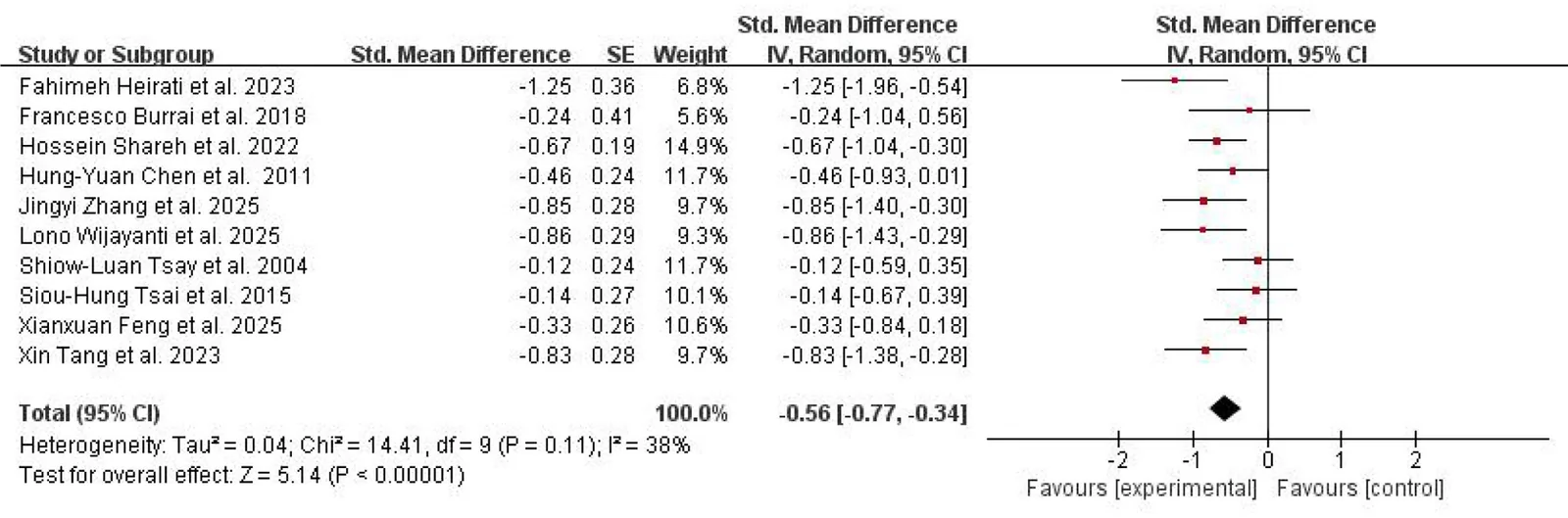
Sensitivity analysis of sleep quality based on 10 studies of non-pharmacological interventions in patients receiving MHD.

Subgroup analyses stratified by intervention type were conducted according to the intervention classification framework described in the Methods section to examine whether the effects on sleep quality differed across non-pharmacological intervention types. The included interventions were categorized into five groups: exercise-based interventions, including combined aerobic-resistance exercise and elastic-band exercise (41, 51); cognitive-behavioral interventions, including cognitive behavioral group therapy and cognitive behavioral therapy (46, 54); music-relaxation interventions, including concert listening intervention and Western music intervention (42, 47); mind-body relaxation interventions, including religion-based spiritual audio intervention, guided imagery therapy, breathing training, and emotional release therapy (44, 45, 48, 49); and complementary and traditional medicine-related interventions, including aromatherapy, foot reflexology massage, and acupressure (43, 50, 52, 53). Within the subgroups, statistically significant improvements in sleep quality were observed for cognitive-behavioral interventions (SMD = −0.59, 95% CI: −0.88 to −0.30, *P* < 0.0001), music-relaxation interventions (SMD = −0.62, 95% CI: −1.20 to −0.04, *P* = 0.04), mind-body relaxation interventions (SMD = −0.67, 95% CI: −1.11 to −0.22, *P* = 0.003), and complementary and traditional medicine-related interventions (SMD = −1.47, 95% CI: −2.60 to −0.34, *P* = 0.01), whereas the exercise-based intervention subgroup did not show a statistically significant improvement (SMD = −0.96, 95% CI: −2.20 to 0.29, *P* = 0.13). High heterogeneity was observed in the exercise-based intervention subgroup (I^2^ = 90%, *P* = 0.001) and the complementary and traditional medicine-related intervention subgroup (I^2^ = 94%, *P* < 0.00001). No statistical heterogeneity was observed in the cognitive-behavioral intervention subgroup (I^2^ = 0%, *P* = 0.49), while moderate heterogeneity was observed in the music-relaxation intervention subgroup (I^2^ = 34%, *P* = 0.22) and the mind-body relaxation intervention subgroup (I^2^ = 45%, *P* = 0.14). Importantly, the test for subgroup differences showed no statistically significant differences in effect sizes among the five intervention types (χ^2^ = 2.42, df = 4, *P* = 0.66, I^2^ = 0%). The overall pooled estimate remained consistent with the primary analysis (Supplementary Figure S3). Subgroup analyses stratified by intervention duration were also conducted. The included studies were divided into short-term interventions ( ≤ 4 weeks) (43, 45, 48–50, 52, 53) and long-term interventions (>4 weeks) (41, 42, 44, 46, 47, 51). The short-term intervention subgroup showed a statistically significant improvement in sleep quality (SMD = −1.16, 95% CI: −1.87 to −0.45, *P* = 0.001), with high heterogeneity (I^2^ = 92%, *P* < 0.00001). The long-term intervention subgroup also showed a statistically significant improvement in sleep quality (SMD = −0.72, 95% CI: −1.02 to −0.41, *P* < 0.00001), with moderate heterogeneity (I^2^ = 57%, *P* = 0.03). Although the pooled effect estimate was numerically larger for short-term interventions, the test for subgroup differences indicated no statistically significant difference between short- and long-term interventions (χ^2^ = 1.28, df = 1, *P* = 0.26, I^2^ = 22.0%). The overall pooled estimate was also consistent with the primary analysis (Supplementary Figure S4). Overall, subgroup analyses based on intervention type and duration did not identify statistically significant differences in effect sizes between subgroups. These findings should be interpreted cautiously because some subgroups included a limited number of RCTs, which may have limited the ability to detect potentially meaningful between-subgroup differences.

### 3.4 Publication bias analysis

A funnel plot was generated for depression-related outcomes (*n* = 9) (Supplementary Figure S5). Visual inspection suggested possible asymmetry. Because fewer than 10 studies were included, Egger's test was not performed. Therefore, the observed asymmetry was interpreted as potential funnel plot asymmetry rather than definitive evidence of publication bias, and the findings should be interpreted cautiously. For anxiety-related outcomes (n = 11) and sleep-related outcomes (n = 14), Egger's linear regression tests were conducted alongside visual inspection of the funnel plots (Supplementary Figures S6–S9). For anxiety, Egger's test yielded t = −1.13, df = 9, and *P* = 0.286, whereas for sleep quality, it yielded t = 0.2342, df = 12, and *P* = 0.8188. Neither analysis showed statistically significant evidence of funnel plot asymmetry. However, the possibility of publication bias could not be completely excluded, as funnel plot asymmetry may be influenced by small-study effects, clinical heterogeneity, and other factors. Therefore, these findings should be interpreted cautiously.

### 3.5 Quality of evidence

Following the GRADE approach, the certainty of evidence was rated as low for sleep quality and anxiety and moderate for depression. The justifications for these ratings and other details are presented in Table 2.

**Table 2:** GRADE summary of the quality of the evidence for the outcomes.

Outcome	Quality assessment						Number of participants (studies)	Effect size, SMD (95%CI)	Quality of the evidence (GRADE^a^)
	Study design	Risk of bias	Inconsistency	Indirectness	Imprecision	Other considerations (Publication bias strongly suspected)			
Sleep quality	randomized trials	Serious^b^	Serious^c^	Not serious^e^	Not serious^f^	Undetected^g^	871 (14)	−0.94 (−1.33, −0.55)	⊕⊕°° Low
Anxiety	randomized trials	Serious^b^	Serious^c^	Not serious^e^	Not serious^f^	Undetected^g^	647 (11)	−0.86 (−1.28, −0.43)	⊕⊕°° Low
Depression	randomized trials	Serious^b^	Not serious^d^	Not serious^e^	Not serious^f^	Undetected^h^	570 (9)	−0.62 (−0.87, −0.37)	⊕⊕⊕°Moderate

^a^GRADE: grading of recommendations, assessment, development, and evaluation.

^b^No study was at overall low risk of bias, and most had some concerns regarding randomization, allocation concealment, blinding, or outcome reporting.

^c^Substantial heterogeneity was observed, although it was reduced in the *post hoc* exploratory sensitivity analysis; residual inconsistency could not be excluded.

^d^Moderate heterogeneity was observed in the main analysis, which was reduced to low heterogeneity in the leave-one-out sensitivity analysis.

^e^The population, interventions, comparators, and outcomes were directly relevant to the research question.

^f^The estimates were reasonably precise, with 95% CIs that did not cross the line of no effect.

^g^No evidence of publication bias was detected by funnel plot inspection and Egger's test.

^h^Funnel plot asymmetry suggested potential publication bias; however, the evidence was insufficient to confirm its presence or warrant downgrading.

## 4 Discussion

This systematic review and meta-analysis included 14 randomized controlled trials involving 871 patients receiving MHD. Pooled analyses suggested that, compared with routine care, non-pharmacological interventions were associated with improvements in depression, anxiety, and sleep quality, with moderate-to-large effect sizes in the main analyses. However, substantial heterogeneity was observed for anxiety and sleep quality. According to the GRADE assessment, the certainty of evidence was moderate for depression and low for anxiety and sleep quality, indicating that the available evidence, particularly for anxiety and sleep quality, remains limited. Importantly, the pooled SMDs represent statistical differences between intervention and control groups and should not be directly interpreted as clinically meaningful improvements at the individual patient level. In addition, the pooled estimates were intended to characterize the overall potential effects of non-pharmacological interventions as a broad intervention category and should not be interpreted as indicating equivalent treatment effects across different intervention modalities or durations. Overall, non-pharmacological interventions may offer potential benefits for psychological symptoms and sleep quality among patients receiving MHD; however, the substantial heterogeneity and limited certainty of evidence warrant cautious interpretation of the pooled findings, particularly for anxiety and sleep quality. Further high-quality randomized controlled trials are warranted to clarify the effectiveness of specific intervention modalities and durations and to establish their clinical relevance and longer-term effects.

### 4.1 Interpretation of the findings

The results of this meta-analysis suggest that non-pharmacological interventions may improve depressive symptoms in patients receiving MHD, consistent with findings from previous systematic reviews (55–57). As an essential component of comprehensive clinical care, non-pharmacological interventions may complement pharmacological treatment by alleviating symptoms, reducing the burden of polypharmacy, and supporting patients' overall physical and psychological wellbeing (58). The potential benefits of these interventions may involve multiple psychological, behavioral, and physiological pathways. Psychological and supportive interventions may help alleviate psychological distress and support emotional wellbeing, whereas exercise and other non-pharmacological approaches may also influence physiological processes relevant to psychological health (59–62). These mechanisms should be considered potential pathways underlying the observed effects, as they were not directly examined in the present meta-analysis. The pooled effect was moderate in magnitude. However, the leave-one-out sensitivity analysis showed that exclusion of study (51) reduced the pooled effect size and decreased heterogeneity from I^2^ = 52% to 38%, suggesting that study may have contributed to the observed heterogeneity. Further comparison of baseline characteristics showed that participants in study had a higher mean age than those in the other included studies, suggesting that differences in age distribution may have contributed to variability in the effect estimates. However, this interpretation is based on a *post-hoc* observation and does not establish an independent effect of age, which cannot be disentangled from other between-study differences. Furthermore, the limited number of studies reporting depressive outcomes precluded subgroup analyses by intervention type, intervention duration, or assessment instrument, thereby limiting further exploration of potential sources of heterogeneity. Although the GRADE assessment indicated moderate-certainty evidence for depressive outcomes, potential publication bias could not be excluded because visual inspection of the funnel plot suggested possible asymmetry. However, the clinical significance of the observed improvement cannot be determined solely from the SMD. Because different depression assessment instruments were used across studies, a common minimal clinically important difference (MCID) cannot be directly applied to the pooled SMD. Therefore, further rigorously designed, adequately powered RCTs are warranted to validate these findings and determine whether the observed improvement translates into clinically meaningful benefits for patients receiving MHD.

The findings of this meta-analysis suggest that non-pharmacological interventions may improve anxiety symptoms in patients receiving MHD, which is broadly consistent with previous research (63). Anxiety is a common psychological problem among patients receiving MHD and frequently co-occurs with depressive symptoms. Persistent anxiety may adversely affect quality of life and contribute to the overall disease-related burden (64). Non-pharmacological interventions may alleviate anxiety through multiple psychological and behavioral pathways, including psychological support, adaptive coping, and relaxation-based strategies (65). These potential pathways may contribute to the observed improvement in anxiety, although their specific mechanisms remain to be clarified. The pooled analysis showed a large effect size for anxiety symptoms; however, substantial heterogeneity was observed across the included studies. Exploratory sensitivity analysis showed that heterogeneity decreased from I^2^ = 79% to 7% after excluding studies (45, 48). Further comparison of study characteristics revealed that study (45) employed a religion-based spiritual audio intervention and had a relatively distinct effect estimate, whereas study (48) used guided imagery relaxation, which was relatively uncommon among the included trials. These differences in intervention characteristics may have contributed in part to the observed heterogeneity. However, these interpretations are *post-hoc* observations and cannot establish a causal relationship. Because only a limited number of studies reported anxiety outcomes, subgroup analyses by intervention type, intervention duration, or assessment instrument could not be performed, limiting further exploration of potential sources of heterogeneity. According to the GRADE assessment, the certainty of evidence for anxiety outcomes was low, indicating some uncertainty in the pooled estimate and suggesting that the true effect may differ substantially from the current estimate. In addition, publication bias for anxiety outcomes was assessed using funnel plot inspection and Egger's test, with no statistically significant evidence of funnel plot asymmetry. However, these findings may be influenced by clinical heterogeneity and methodological differences across studies; therefore, potential publication bias cannot be completely excluded. Therefore, although non-pharmacological interventions may provide benefits for anxiety symptoms in patients receiving MHD, the clinical implications of the observed effect should be interpreted cautiously. Because different anxiety assessment instruments were used across studies, the clinical significance of the pooled SMD cannot be determined directly, and a common MCID cannot be applied to the pooled estimate. Thus, the relatively large pooled effect should not be directly equated with a definite clinically meaningful benefit. In clinical practice, non-pharmacological interventions may be considered as adjunctive supportive strategies, with the choice of intervention tailored to patients' psychological status, physical condition, treatment preferences, and tolerance. Further well-designed, adequately powered randomized controlled trials are warranted to confirm these findings and determine the clinical value and applicability of different non-pharmacological interventions for anxiety management in patients undergoing MHD.

The findings of this meta-analysis suggest that non-pharmacological interventions may improve sleep quality in patients undergoing MHD, which is broadly consistent with previous research (66, 67). Poor sleep quality may be associated with circadian rhythm disruption and adverse outcomes, including fatigue, cognitive impairment, memory decline, and reduced quality of life (68). Non-pharmacological interventions may improve sleep quality by alleviating psychological tension, promoting physical and mental relaxation, and modifying sleep-related cognitive and behavioral patterns (69). These potential pathways may contribute to improvements in sleep quality, although their specific mechanisms remain to be clarified. Notably, sleep quality in the present study was assessed using two instruments, the PSQI and the RCSQ. Although these instruments differ in scoring methods and specific assessment domains, both are established tools for assessing sleep quality (70, 71). To ensure consistency in the direction of interpretation, the scoring direction of the instruments was standardized so that higher scores uniformly indicated poorer sleep quality. The SMD was then used to account for differences in measurement scales and to pool sleep quality outcomes assessed using the two instruments. The pooled analysis showed a relatively large favorable effect estimate; however, substantial heterogeneity was observed across the included studies. Exploratory sensitivity analysis showed that exclusion of studies (41, 43, 48, 50) reduced heterogeneity from I^2^ = 87% to 38%. Further comparison of study chara cteristics indicated that study (41) had a relatively small sample size and employed a combined aerobic and resistance exercise intervention; study (43) assessed sleep quality using the RCSQ rather than the PSQI; study (48) used guided imagery relaxation, which represented a relatively distinct intervention modality in this review; and participants in study (50) may have had different baseline sleep characteristics from those in other studies. Differences in intervention characteristics, outcome assessment instruments, and participant characteristics may have partially contributed to the observed heterogeneity. However, these interpretations were based on *post-hoc* observations and cannot establish causal relationships. Therefore, the findings of the exploratory sensitivity analysis should be interpreted with caution. Subgroup analyses further showed favorable effects of cognitive-behavioral, music-based relaxation, mind–body relaxation, and complementary and traditional medicine-related interventions on sleep quality, which were generally consistent with previous research. Cognitive-behavioral interventions may target maladaptive sleep-related cognitions and behaviors, thereby helping patients establish healthier sleep patterns (72). Music, mind–body relaxation, and complementary and traditional medicine-related interventions may improve sleep quality by promoting relaxation, alleviating psychological distress, and relieving sleep-related discomfort (73–76). In contrast, the exercise subgroup did not show a statistically significant effect, which differed from the findings of some previous meta-analyses (77). This discrepancy may be related to differences in intervention protocols and clinical characteristics, as well as the limited number of studies and small sample size in the exercise subgroup. In addition, variations in exercise modality, intensity, and intervention duration across studies may have contributed to differences in observed intervention effects. The nonsignificant test for subgroup differences does not support the conclusion that any particular intervention modality is superior to others. Therefore, the absence of a statistically significant effect in the exercise subgroup should not be interpreted as evidence that exercise interventions are ineffective in improving sleep quality. With respect to intervention duration, both short- and long-term intervention subgroups showed statistically significant improvements in sleep quality. Although the effect estimate was numerically larger for short-term interventions, the difference between the two subgroups was not statistically significant, and short-term interventions therefore cannot be considered superior to long-term interventions. The greater heterogeneity observed in the short-term subgroup may be related to differences in intervention modalities, implementation frequency, and intervention duration. Given the limited number of studies included in some subgroups, the current evidence remains insufficient to determine the optimal intervention duration for improving sleep quality in patients receiving MHD. According to the GRADE assessment, the certainty of evidence for sleep quality was low, indicating limited confidence in the pooled effect estimate and suggesting that the true effect may differ from the current estimate. In addition, publication bias for the sleep quality outcome was assessed using funnel plot inspection and Egger's test, with no clear statistical evidence of publication bias identified. However, these findings may be influenced by clinical heterogeneity and methodological differences across studies; therefore, potential publication bias cannot be completely excluded, and the results should be interpreted cautiously. From a clinical perspective, the observed improvement suggests that non-pharmacological interventions may have potential value in the management of sleep quality among patients receiving MHD. Improvements in sleep quality may help alleviate sleep-related discomfort and potentially contribute to better daytime functioning and overall wellbeing. However, because the present meta-analysis used SMD to pool outcomes assessed with different sleep quality instruments, and because the PSQI and RCSQ differ in scoring ranges and clinical interpretation, it remains unclear whether the observed improvement meets a patient-perceived MCID. Therefore, the relatively large pooled effect should be interpreted as a favorable statistical effect rather than being directly equated with a definite clinical benefit. Future studies should incorporate instrument-specific clinical thresholds and patient-reported outcomes to further determine the clinical relevance of improvements in sleep quality.

### 4.2 Limitations

#### 4.2.1 Limitations related to heterogeneity and methodological quality

Substantial heterogeneity was observed, particularly for anxiety and sleep quality, potentially reflecting differences in participant characteristics, intervention modalities and durations, measurement instruments, and outcome assessment methods. These sources of heterogeneity may have contributed to variations in the observed intervention effects and should therefore be considered when interpreting the pooled effect estimates. Exploratory subgroup analyses for sleep quality were conducted by intervention modality and duration; however, the limited number of studies in some subgroups may have reduced statistical power to detect meaningful between-group differences. Further subgroup analyses for depression and anxiety were not feasible because of the limited number of eligible studies. A *post-hoc* multiple-study exclusion sensitivity analysis suggested that some studies may have contributed to the heterogeneity observed for anxiety and sleep quality; however, as this analysis was not pre-specified, these findings should be interpreted cautiously and cannot establish the specific sources of heterogeneity.

Methodological limitations were also present. Twelve of the 14 trials had some concerns regarding overall risk of bias, while two had a high risk of bias. Incomplete reporting of allocation concealment and the inherent difficulty of blinding participants and intervention providers in non-pharmacological trials may have introduced potential bias and reduced confidence in the pooled estimates. These limitations, together with the observed heterogeneity, contributed to uncertainty in the findings and were reflected in the GRADE assessment.

#### 4.2.2 Limitations related to literature search and generalizability

The review was restricted to peer-reviewed studies published in English and excluded non-English publications and gray literature, potentially introducing language and publication bias. The predominance of studies conducted in Asian countries may also limit the generalizability of the findings to other geographic and healthcare settings. These restrictions may have limited the comprehensiveness and representativeness of the evidence and potentially influenced the pooled estimates, although the direction and magnitude of such effects remain uncertain. Future reviews should consider evidence from multiple languages, relevant gray literature, and diverse geographic and healthcare settings.

## 5 Conclusion

This meta-analysis of 14 randomized controlled trials suggests that non-pharmacological interventions may improve depression, anxiety, and sleep quality in patients undergoing maintenance hemodialysis. Given the heterogeneity and methodological limitations of the included studies, these findings should be interpreted cautiously. The certainty of evidence was moderate for depression and low for anxiety and sleep quality according to the GRADE assessment. Thus, non-pharmacological interventions may be considered supportive or adjunctive strategies, but the current evidence is insufficient to support strong clinical recommendations. Further well-designed, adequately powered randomized controlled trials with standardized interventions and longer follow-up are warranted to confirm these findings and clarify their clinical relevance.

## Data Availability

The original contributions presented in the study are included in the article/Supplementary material, further inquiries can be directed to the corresponding author.
