# Supplementary material for: A systematic review and meta-analysis of the effects of non-pharmacological interventions in improving psychological status and sleep quality among hemodialysis patients
Source: Front Public Health. 2026 Sep 28;14:1919741. doi: 10.3389/fpubh.2026.1919741 (PMC13655104; doi:10.3389/fpubh.2026.1919741)
Supplement: Supplementary file 1 [file Table_1.docx]

Supplementary Material

Table S1. Eligibility verification of included studies based on the PICOS criteria

| **Included studies** | **Population** | **Interventions** | **Control interventions** | **Outcomes** | **Study design** |
| --- | --- | --- | --- | --- | --- |
| Li A et al. 2025[41] | patients who underwent maintenance hemodialysis combined with RLS | Aerobic combined with resistance exercise | Usual care | SDS, SAS, PSQI | RCT |
| Zhang JY et al.2025[42] | patients undergoing maintenance hemodialysis | Western music | Usual care | HADS-D, HADS-A, PSQI | RCT |
| Göktuna G et al. 2024[43] | patients undergoing hemodialysis treatment | Foot reflexology massage | Usual care | STAI-S, RCSQ | RCT |
| Tang X et al.2023[44] | patients with end‑stage renal disease receiving maintenance hemodialysis | Emotional Freedom Techniques | Usual care | HADS-D, HADS-A, PSQI | RCT |
| Wijayanti L et al. 2025[45] | hemodialysis patients | Combined audio therapy | Usual care | HARS, PSQI | RCT |
| Shareh H et al.2022[46] | patients undergoing haemodialysis | Group cognitive behavioral therapy for insomnia | Usual care | BDI-II, BAI, PSQI | RCT |
| Burrai F et al.2018[47] | patients with a diagnosis of end‑stage kidney disease undergoing hemodialysis | Live singing | Usual care | HADS-D, HADS-A, PSQI | RCT |
| Afshar M et al. 2018[48] | hemodialysis patients | Guided imagery | Usual care | STAI-S, PSQI | RCT |
| Tsai SH et al.2015[49] | patients with CKD who were on maintenance hemodialysis | Breathing training group | Waitlist | BDI-II, PSQI | RCT |
| Suandika M et al. 2023[50] | patients with end‑stage renal disease receiving hemodialysis treatment | True acupressure | Usual care | HADS-D, HADS-A, PSQI | RCT |
| Feng X et al.2025[51] | patients on maintenance hemodialysis | Resistance band exercise | Usual care | HAMD, HAMA, PSQI | RCT |
| Tsay SL et al.2004[52] | patients with end‑stage renal disease | Acupressure | Usual care | BDI, PSQI | RCT |
| Heirati F et al.2023[53] | patients undergoing dialysis | Aromatherapy group | Usual care | STAI-S, PSQI | RCT |
| Chen HY et al. 2011[54] | patients undergoing maintenance hemodialysis | Cognitive behavioral therapy | Usual care | BDI, BAI, PSQI | RCT |

Table S2 Bias risk assessment table.

| **Included studies** | **D1** | **D2** | **D3** | **D4** | **D5** | **All** |
| --- | --- | --- | --- | --- | --- | --- |
| Li A et al. 2025[41] | Some concerns | Some concerns | Low risk | Some concerns | Some concerns | Some concerns |
| Zhang JY et al.2025[42] | Low risk | Some concerns | Low risk | Some concerns | Low risk | Some concerns |
| Göktuna G et al. 2024[43] | Some concerns | Some concerns | Some concerns | Some concerns | Some concerns | Some concerns |
| Tang X et al.2023[44] | Some concerns | Some concerns | Some concerns | Some concerns | Some concerns | Some concerns |
| Wijayanti L et al. 2025[45] | Some concerns | Some concerns | Low risk | Some concerns | Some concerns | Some concerns |
| Shareh H et al.2022[46] | Some concerns | High risk | Some concerns | Some concerns | Some concerns | High risk |
| Burrai F et al.2018[47] | Low risk | Some concerns | Low risk | Some concerns | Some concerns | Some concerns |
| Afshar M et al. 2018[48] | Low risk | Some concerns | Low risk | Some concerns | Some concerns | Some concerns |
| Tsai SH et al.2015[49] | Low risk | Some concerns | Some concerns | Some concerns | Some concerns | Some concerns |
| Suandika M et al. 2023[50] | Some concerns | Some concerns | Low risk | Some concerns | Some concerns | Some concerns |
| Feng X et al.2025[51] | Some concerns | Some concerns | Low risk | Low risk | Some concerns | Some concerns |
| Tsay SL et al.2004[52] | Some concerns | Some concerns | Some concerns | High risk | Some concerns | High risk |
| Heirati F et al.2023[53] | Some concerns | Some concerns | Some concerns | Some concerns | Some concerns | Some concerns |
| Chen HY et al. 2011[54] | Low risk | Some concerns | Low risk | Some concerns | Some concerns | Some concerns |

Note: D1: Bias arising from the randomization process; D2Bias due to deviations from intended interventions; D3: Bias due to missing outcome data; D4: Bias in measurement of the outcome; D5：Bias in selection of the reported result.

**Figure S1.** Results of the RoB 2 assessment for 14 randomized controlled trials.

**Figure S2.** Summary plot of risk of bias across the included randomized controlled trials using the RoB 2 tool.

Table S3. Results of leave-one-out sensitivity analysis for depression outcomes

| **Outcome** | **Study removed** | **Pooled SMD (95% CI)** | **I² (%)** |
| --- | --- | --- | --- |
| depressive | Li A et al. 2025[41] | -0.62[-0.90,-0.33] | 58% |
| depressive | Zhang JY et al.2025[42]] | -0.59[-0.86,-0.31] | 55% |
| depressive | Tang X et al.2023[44] | -0.54[-0.77,-0.31] | 38% |
| depressive | Shareh H et al.2022[46] | -0.63[-0.92,-0.33] | 58% |
| depressive | Burrai F et al.2018[47] | -0.64[-0.91,-0.37] | 57% |
| depressive | Tsai SH et al.2015[49] | -0.62[-0.90,-0.68] | 58% |
| depressive | Feng X et al.2025[51] | -0.56[-0.82,-0.31] | 49% |
| depressive | Tsay SL et al.2004[52] | -0,69[-0.93,-0.45] | 41% |
| depressive | Chen HY et al. 2011[54 | -0.68[-0.93,-0.42] | 46% |

Table S4. Results of leave-one-out sensitivity analysis for anxiety outcomes

| **Outcome** | **Study removed** | **Pooled SMD (95% CI)** | **I² (%)** |
| --- | --- | --- | --- |
| anxiety | Li A et al. 2025[41] | -0.88[-1.29,-0.47] | 81% |
| anxiety | Zhang JY et al.2025[42] | -0.88[-1.29,-0.47] | 81% |
| anxiety | Göktuna G et al. 2024[43] | -0.84[-1.24,-0.44] | 81% |
| anxiety | Tang X et al.2023[44] | -0.84[-1.24,-0.44] | 81% |
| anxiety | Wijayanti L et al. 2025[45] | -0.78[-1.15,-0.41] | 78% |
| anxiety | Shareh H et al.2022[46] | -0.90[-1.32,-0.48] | 81% |
| anxiety | Burrai F et al.2018[47] | -0.90[-1.29,-0.51] | 81% |
| anxiety | Suandika M et al. 2023[50] | -0.70[-0.96,-0.44] | 54% |
| anxiety | Feng X et al.2025[51] | -0.90[-1.30,-0.50] | 81% |
| anxiety | Heirati F et al.2023[53] | -0.92[-1.31,-0.52] | 80% |
| anxiety | Chen HY et al. 2011[54] | -0.93[-1.32,-0.54] | 79% |

Table S5. Results of leave-one-out sensitivity analysis for anxiety Sleep quality

| **Outcome** | **Included studies** | **Pooled SMD (95% CI)** | **I² (%)** |
| --- | --- | --- | --- |
| Sleep quality | Li A et al. 2025 [41] | -0.89[-1.30,-0.48] | 88% |
| Sleep quality | Zhang JY et al.2025 [42] | -0.95[-1.37,-0.53] | 88% |
| Sleep quality | Göktuna G et al. 2024 [43] | -0.85[-1.23,-0.46] | 86% |
| Sleep quality | Tang X et al.2023 [44] | -0.95[-1.37,-0.53] | 88% |
| Sleep quality | Wijayanti L et al. 2025[45] | -0.95[-1.37,-0.53] | 88% |
| Sleep quality | Shareh H et al.2022[46] | -0.96[-1.40,-0.53] | 88% |
| Sleep quality | Burrai F et al.2018 [47] | -0.99[-1.39,-0.58] | 88% |
| Sleep quality | Afshar M et al. 2018 [48] | -0.91[-1.33,-0.49] | 88% |
| Sleep quality | Tsai SH et al.2015 [49] | -1.00[-1.41,-0.60] | 87% |
| Sleep quality | Suandika M et al. 2023 [50] | -0.82[-1.13,-0.51] | 76% |
| Sleep quality | Feng X et al.2025 [51] | -0.99[-1.40,-0.58] | 87% |
| Sleep quality | Tsay SL et al.2004 [52] | -1.01[-1.41,-0.61] | 86% |
| Sleep quality | Heirati F et al.2023 [53] | -0.92[-1.33,-0.5] | 88% |
| Sleep quality | Chen HY et al. 2011 [54] | -0.98[-1.40,-0.56] | 88% |

**Figure S3.** Subgroup analysis of sleep quality of the 14 included studies of non-pharmacological interventions for MHD patients (subgrouped by intervention type)

**Figure S4.** Subgroup analysis of sleep quality of the 14 included studies of non-pharmacological interventions for MHD patients (stratified by intervention type)

**Figure S5.** Funnel plot of non-pharmacological interventions for improving depressive symptoms in MHD patients.

**Figure S6.** Egger regression plot to assess publication bias of non-pharmacological interventions for anxiety symptoms in MHD patients.

**Figure S7.** Funnel plot of non-pharmacological interventions for improving anxiety symptoms in MHD patients.

**Figure S8.** Egger regression plot to assess publication bias of non-pharmacological interventions for sleep quality in MHD patients.

**Figure S9**. Funnel plot of non-pharmacological interventions for improving sleep quality in MHD patients.
