# Supplementary material for: A systematic review and meta-analysis of the effects of non-pharmacological interventions in improving psychological status and sleep quality among hemodialysis patients
Source: Front Public Health. 2026 Sep 28;14:1919741. doi: 10.3389/fpubh.2026.1919741 (PMC13655104; doi:10.3389/fpubh.2026.1919741)
Supplement: Supplementary file 2 [file Table_2.docx]

Supplementary Material

**PubMed**

("Renal Dialysis" OR Renal Dialys* OR Extracorporeal Dialys* OR Hemodialys* OR haemodialysis OR HD OR "blood dialysis" OR "maintenance hemodialysis" OR "chronic hemodialysis" OR "renal replacement therapy")

AND

(Napping OR "Nighttime Sleep" OR sleeping OR sleep quality OR sleep disturbance OR sleep disorder OR insomnia OR sleep)

AND

(Depression OR "Depressive Symptoms" OR "Emotional Depression" OR Anxiety Disorder* OR "Neurotic Anxiety State" OR Anxiety Neuroses OR Psychology OR Psychological Factor* OR "Psychological Side Effect*" OR "Mental Health" OR "Mental Disorders" OR Psychiatry OR "Depression, Major" OR Dysthymia OR Apprehension)

AND

("Randomized Controlled Trial" OR "Controlled Clinical Trial" OR RCT)

NOT

(pharmacological treatment OR drug* OR medication OR "antidepressant drugs" OR "anxiolytic drugs" OR "anti-anxiety drugs" OR "sleep medications" OR "hypnotic drugs" OR antidepressants OR "antidepressant preparations" OR thymic stimulants OR Pharmacologies OR Pharmaceutic* Preparation* OR "Pharmaceutical Product*" OR Pharmaceutical* OR Antidepressive Agent* OR Thymoleptic* OR Thymoanaleptic* OR "Medication, Antidepressant" OR Antidepressant OR Antidepressant Drug* OR "Anti-Anxiety Agent*" OR "Anti-Anxiety Effect*" OR "Agents, Minor Tranquillizing" OR "Anti Anxiety Drug*" OR "Minor Tranquilizing Agents" OR "Anti Anxiety Agent" OR Anxiolytic Effect* OR Antianxiety Effect* OR Hypnotic* OR Sedative* OR "Hypnotic Effects" OR Hypnotic Effect* OR "Effects, Sedative" OR Hypnotics OR Hypnotic)

**Embase**

(exp hemodialysis OR Renal Dialys$ OR Extracorporeal Dialys$ OR Hemodialys$ OR haemodialysis OR HD OR 'blood dialysis' OR 'maintenance hemodialysis' OR 'chronic hemodialysis' OR 'renal replacement therapy' OR MHD)

AND

(Napping OR 'Nighttime Sleep' OR sleeping OR sleep quality OR sleep disturbance OR sleep disorder OR insomnia OR sleep)

AND

(Depression OR 'Depressive Symptoms' OR 'Emotional Depression' OR Anxiety Disorder$ OR 'Neurotic Anxiety State' OR Anxiety Neuroses OR Psychology OR Psychological Factor$ OR 'Psychological Side Effect$' OR 'Mental Health' OR 'Mental Disorders' OR Psychiatry OR 'Depression, Major' OR Dysthymia OR Apprehension)

AND

("Randomized Controlled Trial" OR "Controlled Clinical Trial" OR RCT)

NOT

(pharmacological treatment OR drug$ OR medication OR 'antidepressant drugs' OR 'anxiolytic drugs' OR 'anti-anxiety drugs' OR 'sleep medications' OR 'hypnotic drugs' OR antidepressants OR 'antidepressant preparations' OR thymic stimulants OR Pharmacologies OR Pharmaceutic$ Preparation$ OR 'Pharmaceutical Product$' OR Pharmaceutical$ OR Antidepressive Agent$ OR Thymoleptic$ OR Thymoanaleptic$ OR 'Medication, Antidepressant' OR Antidepressant OR Antidepressant Drug$ OR 'Anti-Anxiety Agent$' OR 'Anti-Anxiety Effect$' OR 'Agents, Minor Tranquillizing' OR 'Anti Anxiety Drug$' OR 'Minor Tranquilizing Agents' OR 'Anti Anxiety Agent' OR Anxiolytic Effect$ OR Antianxiety Effect$ OR Hypnotic$ OR Sedative$ OR 'Hypnotic Effects' OR Hypnotic Effect$ OR 'Effects, Sedative' OR Hypnotics OR Hypnotic)

**Web of science**

TS=(Renal Dialys* OR Extracorporeal Dialys* OR Hemodialys* OR haemodialysis OR HD OR "blood dialysis" OR "maintenance hemodialysis" OR "chronic hemodialysis" OR "renal replacement therapy")

AND

TS=(Napping OR "Nighttime Sleep" OR sleeping OR sleep quality OR sleep disturbance OR sleep disorder OR insomnia OR sleep)

AND

TS=(Depression OR "Depressive Symptoms" OR "Emotional Depression" OR Anxiety Disorder* OR "Neurotic Anxiety State" OR Anxiety Neuroses OR Psychology OR Psychological Factor* OR "Psychological Side Effect*" OR "Mental Health" OR "Mental Disorders" OR Psychiatry OR "Depression, Major" OR Dysthymia OR Apprehension)

AND

TS=("Randomized Controlled Trial" OR "Controlled Clinical Trial" OR RCT)

NOT

TS=(pharmacological treatment OR drug* OR medication OR "antidepressant drugs" OR "anxiolytic drugs" OR "anti-anxiety drugs" OR "sleep medications" OR "hypnotic drugs" OR antidepressants OR "antidepressant preparations" OR thymic stimulants OR Pharmacologies OR Pharmaceutic* Preparation* OR "Pharmaceutical Product*" OR Pharmaceutical* OR Antidepressive Agent* OR Thymoleptic* OR Thymoanaleptic* OR "Medication, Antidepressant" OR Antidepressant OR Antidepressant Drug* OR "Anti-Anxiety Agent*" OR "Anti-Anxiety Effect*" OR "Agents, Minor Tranquillizing" OR "Anti Anxiety Drug*" OR "Minor Tranquilizing Agents" OR "Anti Anxiety Agent" OR Anxiolytic Effect* OR Antianxiety Effect* OR Hypnotic* OR Sedative* OR "Hypnotic Effects" OR Hypnotic Effect* OR "Effects, Sedative" OR Hypnotics OR Hypnotic)

**Cochrane Library**

(Renal Dialys* OR Extracorporeal Dialys* OR Hemodialys* OR haemodialysis OR HD OR "blood dialysis" OR "maintenance hemodialysis" OR "chronic hemodialysis" OR "renal replacement therapy")

AND

(Napping OR "Nighttime Sleep" OR sleeping OR sleep quality OR sleep disturbance OR sleep disorder OR insomnia OR sleep)

AND

(Depression OR "Depressive Symptoms" OR "Emotional Depression" OR Anxiety Disorder* OR "Neurotic Anxiety State" OR Anxiety Neuroses OR Psychology OR Psychological Factor* OR "Psychological Side Effect*" OR "Mental Health" OR "Mental Disorders" OR Psychiatry OR "Depression, Major" OR Dysthymia OR Apprehension)

AND

("Randomized Controlled Trial" OR "Controlled Clinical Trial" OR RCT)

NOT

(pharmacological treatment OR drug* OR medication OR "antidepressant drugs" OR "anxiolytic drugs" OR "anti-anxiety drugs" OR "sleep medications" OR "hypnotic drugs" OR antidepressants OR "antidepressant preparations" OR thymic stimulants OR Pharmacologies OR Pharmaceutic* Preparation* OR "Pharmaceutical Product*" OR Pharmaceutical* OR Antidepressive Agent* OR Thymoleptic* OR Thymoanaleptic* OR "Medication, Antidepressant" OR Antidepressant OR Antidepressant Drug* OR "Anti-Anxiety Agent*" OR "Anti-Anxiety Effect*" OR "Agents, Minor Tranquillizing" OR "Anti Anxiety Drug*" OR "Minor Tranquilizing Agents" OR "Anti Anxiety Agent" OR Anxiolytic Effect* OR Antianxiety Effect* OR Hypnotic* OR Sedative* OR "Hypnotic Effects" OR Hypnotic Effect* OR "Effects, Sedative" OR Hypnotics OR Hypnotic)

**CINAHL Complete**

(MH "Renal Dialysis") OR Renal Dialys* OR Extracorporeal Dialys* OR Hemodialys* OR haemodialysis OR HD OR "blood dialysis" OR "maintenance hemodialysis" OR "chronic hemodialysis" OR "renal replacement therapy"

AND

(Napping OR "Nighttime Sleep" OR sleeping OR sleep quality OR sleep disturbance OR sleep disorder OR insomnia OR sleep)

AND

(Depression OR "Depressive Symptoms" OR "Emotional Depression" OR Anxiety Disorder* OR "Neurotic Anxiety State" OR Anxiety Neuroses OR Psychology OR Psychological Factor* OR "Psychological Side Effect*" OR "Mental Health" OR "Mental Disorders" OR Psychiatry OR "Depression, Major" OR Dysthymia OR Apprehension)

AND

("Randomized Controlled Trial" OR "Controlled Clinical Trial" OR RCT)

NOT

(pharmacological treatment OR drug* OR medication OR "antidepressant drugs" OR "anxiolytic drugs" OR "anti-anxiety drugs" OR "sleep medications" OR "hypnotic drugs" OR antidepressants OR "antidepressant preparations" OR thymic stimulants OR Pharmacologies OR Pharmaceutic* Preparation* OR "Pharmaceutical Product*" OR Pharmaceutical* OR Antidepressive Agent* OR Thymoleptic* OR Thymoanaleptic* OR "Medication, Antidepressant" OR Antidepressant OR Antidepressant Drug* OR "Anti-Anxiety Agent*" OR "Anti-Anxiety Effect*" OR "Agents, Minor Tranquillizing" OR "Anti Anxiety Drug*" OR "Minor Tranquilizing Agents" OR "Anti Anxiety Agent" OR Anxiolytic Effect* OR Antianxiety Effect* OR Hypnotic* OR Sedative* OR "Hypnotic Effects" OR Hypnotic Effect* OR "Effects, Sedative" OR Hypnotics OR Hypnotic)
